# Changes of brain-derived neurotrophic factor (BDNF) levels after different exercise protocols: a systematic review of clinical studies in Parkinson’s disease

**DOI:** 10.3389/fphys.2024.1352305

**Published:** 2024-02-20

**Authors:** Andrea Paterno, Giovanni Polsinelli, Bruno Federico

**Affiliations:** Department of Human Sciences, Society and Health, University of Cassino and Southern Lazio, Cassino, Italy

**Keywords:** brain-derived neurotrophic factor, exercise, systematic review, Parkinson’s disease, Metabolic Equivalent of Task

## Abstract

**Background:** Brain-Derived Neurotrophic Factor (BDNF) serum levels are reduced in patients with Parkinson’s Disease (PD).

**Objectives:** This study aimed to assess the effect of exercise intensity, volume and type on BDNF levels in patients with PD.

**Methods:** We searched clinicaltrials.gov, CINAHL, Embase, PubMed, Scopus, Web of Science for both controlled and non-controlled studies in patients with PD, published between 2003 and 2022, which assessed Brain-Derived Neurotrophic Factor before and after different exercise protocols. Exercise intensity was estimated using a time-weighted average of Metabolic Equivalent of Task (MET), while exercise volume was estimated by multiplying MET for the duration of exercise. Exercise types were classified as aerobic, resistance, balance and others. We computed two distinct standardized measures of effects: Hedges’ g to estimate differences between experimental and control group in pre-post intervention BDNF changes, and Cohen’s d to measure pre-post intervention changes in BDNF values for each study arm. Meta-regression and linear regression were used to assess whether these effect measures were associated with intensity, volume and type. PROSPERO registration number: CRD42023418629.

**Results:** Sixteen studies (8 two-arm trials and 8 single-arm trials) including 370 patients with PD were eligible for the systematic review. Selected studies had a large variability in terms of population and intervention characteristics. The meta-analysis showed a significant improvement in BDNF levels in the exercise group compared to the control group, Hedges’ g = 0.70 (95% CI: 0.03, 1.38), with substantial heterogeneity (I^2^ = 76.0%). Between-group differences in intensity were positively associated with change in BDNF in a subset of 5 controlled studies. In the analysis which included non-controlled studies, intensity and total exercise volume were both positively associated with BDNF change. No difference was found according to exercise type.

**Conclusion:** Exercises of greater intensity may increase BDNF levels in patients with PD, while the role of volume of exercise needs to be further explored.

## Introduction

About 6 million individuals are affected by Parkinson’s Disease (PD) worldwide and more than 200,000 people died from this condition in 2016 (GBD 2016 Parkinson’s Disease Collaborators, 2018). PD, which is associated with loss of dopaminergic neurons in the substantia nigra (Fearnley and Lees, 1991), is typically characterized by both motor symptoms, such as bradykinesia, rigidity, rest tremor and postural instability and non-motor features, including cognitive impairment and depression (Lee and Gilbert, 2016). First-line treatment of PD is based on levodopa and other dopamine agonists and it is directed at symptoms, but no therapy can slow down the progression of PD (Bloem et al., 2021).

Physical exercise (i.e., a structured exercise program (Caspersen et al., 1985)) is a non-pharmacological intervention which is a major component of physiotherapy for management of PD (Bouca-Machado et al., 2020) and is often adopted as part of an integrated therapeutic approach (Bloem et al., 2021). In an observational cohort study, patients with PD who exercised regularly, that is more than 150 min/week, showed better physical function and less cognitive decline after 1 year compared to sedentary patients and those who exercised less frequently (Caspersen et al., 1985). Exercise can improve bone metabolism (Amato et al., 2022), balance and walking ability, motor symptoms (bradykinesia, gait and turning) and non-motor symptoms (cognitive deficits, sleep disorders, mood disturbances, and sensory abnormalities) (Xu et al., 2019). In addition, patients may need smaller drug doses, because the effect of levodopa is often improved by exercise (Muhlack et al., 2007).

Although many types of physical exercise can help improve movement and quality of life for people with PD, there is no evidence that certain exercise types work better than others (Ernst et al., 2023). NICE recommends physiotherapy and occupational therapy for patients who experience difficulties in motor function and daily living activities, without specifying the type of exercise (Rogers et al., 2017). Similarly, European physiotherapy guidelines recommend that patients reduce their daily sitting time and exercise at least 150 min/week, according to their own preferences and physical capabilities (Keus et al., 2014).

It was suggested that aerobic training (AT) improves motor function, although long-term effects are not clear (Schootemeijer et al., 2020). Other studies reported that Nordic walking, dance, cycling, Tai Chi and Qigong and walking improve physical fitness and mobility (Song et al., 2017; Tiihonen et al., 2021; Peyre-Tartaruga et al., 2022). It was also reported that resistance training (RT) improves physical function and quality of life in PD (Gollan et al., 2022). The American College of Sports Medicine recommends that patients with PD engage in Balance Training (BT) in addition to the regular practice of AT and RT (American College of Sports Medicine et al., 2020). A recent meta-analysis of 109 trials showed that dancing was superior to other types of exercise in improving motor function, whereas Nordic walking and Qigong were the most effective exercises that improved mobility and manual dexterity, respectively (Zhang et al., 2023).

The effect of exercise on these clinical outcomes can be explained by a variety of mechanisms (Xu et al., 2019), including the synthesis of several neurotrophic factors, as reported in a review of animal studies and in a systematic review in patients with PD (da Silva et al., 2016; Li et al., 2023). Brain-Derived Neurotrophic Factor (BDNF) protects the brain against destruction of dopaminergic neurons and acts as a growth factor for dopaminergic neurons of the substantia nigra pars compacta (Hyman et al., 1991). Patients with PD have decreased serum levels of BDNF compared to healthy individuals (Rahmani et al., 2019); furthermore, severity of PD symptoms is inversely associated with BDNF concentration (Scalzo et al., 2010).

It is known that regular exercise impacts resting BDNF levels in healthy subjects, with a moderate effect size (Szuhany et al., 2015; Hyman et al., 1991). A meta-analysis of exercise programs in older adults showed that strength training significantly increased BDNF concentration, while AT did not exert such an effect (Marinus et al., 2019). Another systematic review reported that the increase in BDNF concentrations was larger after AT compared to RT (Dinoff et al., 2016), whereas a recent network meta-analysis found that changes in BDNF were greater after RT than AT, but results were pooled from mixed populations, with only a minority of studies recruiting PD patients (Zhou et al., 2022).

There is uncertainty not only about the type of exercise that should be recommended to patients with PD, but also regarding its frequency and intensity (Martignon et al., 2021; Cui et al., 2023; El Hayek et al., 2023). A synthesis of exercise guidelines suggests that patients with mild to moderate PD should engage in three to five sessions of AT at moderate intensity (40%–60% maximum heart rate) per week and two to three sessions of RT, but the evidence base is less consistent compared to other neurological conditions (Kim et al., 2019). A high intensity (>75% maximum heart rate for AT and >70% 1-repetition maximum for RT) is needed according to some scholars but not others (Machado et al., 2022; Rotondo et al., 2023). Recently, a feasible, safe and accessible home-based high-intensity program was proposed (Harpham et al., 2023). It has also been suggested that the health benefits of physical activity are linked to the total amount of exercise (volume), rather than each component (intensity, type, frequency) (Pate et al., 1995). A meta-analysis of observational studies showed an inverse association between weekly physical activity volume and risk of developing PD in men (Fang et al., 2018).

Previous research, including recent systematic reviews on the effect of exercise on BDNF levels, limited the analysis to controlled trials only (Hirsch et al., 2018; Li et al., 2023; Rotondo et al., 2023). Randomised trials provide evidence of efficacy of interventions in “ideal” settings, whereas non-randomised studies more accurately reflect usual clinical practice (Sørensen et al., 2006). Furthermore, exercise interventions in PD are so heterogeneous that they were classified into 18 different combinations of duration, intensity and type (Zhou et al., 2022; Rotondo et al., 2023). This heterogeneity, coupled with a very limited number of trials, makes the evaluation of the effectiveness of exercise intervention extremely hard. Recent meta-analyses aimed at assessing whether exercise determined an improvement of BDNF levels (Hirsch et al., 2018; Li et al., 2023; Rotondo et al., 2023), but a quantitative estimate of the effect of exercise intensity and volume is still lacking. Since it is not known which characteristics of exercise determine the largest benefits for patients with PD, this study aimed at assessing to what extent intensity, volume and type of exercise are associated with changes in BDNF levels in patients with PD.

## Methods

We used the methods proposed in the Preferred Reporting Items for Systematic Reviews and Meta-Analyses (PRISMA) guidelines (Page et al., 2021). This systematic review and meta-analysis was registered in the International Prospective Register of Systematic Reviews (PROSPERO) with the protocol number CRD42023418629, which is available online at https://www.crd.york.ac.uk/prospero/display_record.php?RecordID=418629.

### Search strategy and selection criteria

We searched clinicaltrials.gov, CINHAL, Embase, PubMed, Scopus, Web of Science for studies listed from 1 January 2003 to 31 December 2022. A further literature review update was performed in May 2023.

The following string of terms was used: (Parkinson*) AND (exercise OR “physical activity” OR training OR sport* OR rehabilit* OR “physical therapy” OR physiotherapy) AND (BDNF OR plastic* OR synap* OR neuro* OR cognit* OR biomarker*) NOT (rat OR animal OR mouse OR mice) NOT review. Supplementary Material S1 details the string for each bibliographic database. Reference lists of eligible studies were manually examined for further identification of relevant articles.

### Data extraction

After removing duplicates and reviewing the title and abstract of potential studies, we systematically assessed the full text of identified manuscripts for eligibility. The following data were extracted by two authors (AP and GP) for each study and study arm:

(1) study characteristics (title, authors and year of publication, type of study, sample size);
(2) participants’ information (age, sex, diagnosis, disease duration (years), disease stage (Hoehn and Yahr), motor examination (MDS-UPDRS part 3), pharmacological treatment;
(3) characteristics of the exercise protocol (duration in weeks, number of weekly sessions, duration of each exercise session, description of exercise);
(4) biological sample examined, method of analysis, mean and standard deviation of BDNF at baseline and at the end of the exercise protocol (at least 12 h after the final exercise session). If more than two measures of BDNF were reported after the training, the measurement closer in time to the end of the training was chosen. Supplementary Material S2 describes the methods of BDNF measurement for each study.

Any discrepancies in data extraction were resolved by reference to the original article and discussion between the researchers. If the two authors reached no consensus, a third author (BF) made the final judgement. In case of doubts, we asked the original investigators for additional data and clarification of methods. If the response was unsatisfactory, we extracted relevant data from a previous review (Li et al., 2023).

To be eligible for inclusion, studies had to meet the following criteria: 1) they recruited human participants with diagnosis of PD; 2) they used an experimental design with or without a control group; 3) they contained physical exercise training; 4) they assessed BDNF before and after the exercise protocol. Study protocols, review articles and observational studies were excluded. We also excluded study arms (but not studies) that contained, in addition to the exercise interventions, other interventions that were not exercise-based, such as diet or other techniques, such as transcranial magnetic stimulation (Aftanas et al., 2018; Oliveira et al., 2020).

### Study quality assessment

To assess the risk of bias of randomized trials, the Cochrane Risk of Bias tool was used (Higgins et al., 2011). Risk of bias was assessed within seven domains: 1) random sequence generation, 2) allocation concealment, 3) blinding of participants and personnel, 4) blinding of outcome assessment, 5) incomplete outcome data, 6) selective reporting, 7) other sources of bias. The Risk Of Bias In Non-randomized Studies of Interventions (ROBINS-I) tool was used to assess the risk of bias for non-randomized studies (Sterne et al., 2016). This tool includes seven domains: 1) bias due to confounding, 2) bias in selection of participants into the study, 3) bias in classification of interventions, 4) bias due to deviations from intended interventions, 5) bias due to missing data, 6) bias in measurement of outcomes, and 7) bias in selection of the reported results. These assessments were performed independently by two reviewers (AP, BF). If the two authors reached no consensus, a third reviewer (GP) made the final judgement.

### Data-analysis

Intensity of exercise was estimated with the use of the Metabolic Equivalent of Task (MET), a measure which describes the energy expenditure of a specific activity relative to a rest state (Committee, 2008). Firstly, we chose the MET values of the activity that best matched the exercise described in the study protocol using ACSM conversion tables and Ainsworth compendium (Ainsworth et al., 2011; Garber et al., 2011). Secondly, since exercise interventions often comprised multiple exercise activities, we computed a time-weighted average of MET values (Gomersall et al., 2011). Inactive control groups under routine or usual care that did not follow a specific exercise protocol, were assigned MET = 1.

The total volume of exercise (expressed in MET-hours) was calculated by multiplying the average weekly training volume (in MET-hours/week) for the duration in weeks of the exercise protocol. For each exercise activity, the assigned MET value was multiplied by the duration in minutes of the training session and by the number of weekly sessions. When an exercise protocol included more than one activity, MET-hours/week of each activity were summed up. Volume of physical activity (in MET-hours) was calculated in a previous observational cohort study in a similar manner (Yang et al., 2015). We also calculated the proportion of volume spent practicing different types of exercise (aerobic, resistance, balance and other) (American College of Sports Medicine et al., 2020); the type with the largest volume was used to define the “dominant” type of each exercise protocol.

We computed two distinct standardized measures of effects. In the first analysis (controlled studies only), we computed Hedges’ g to estimate the standardized mean difference (SMD) between experimental and control group in pre-post intervention BDNF changes (Egger et al., 2022). When mean and standard deviation of BDNF were not available in the original studies we derived Hedges’ g from a previous review (Li et al., 2023). In the second analysis, which included both controlled and non-controlled studies, we computed a measure of pre-post intervention change in BDNF values for each study arm, calculating Cohen’s d (Egger et al., 2022).

We conducted a random-effects meta-analysis to obtain a summary estimate of the effect of exercise interventions. The I^2^ statistic was used to assess inconsistency between studies. In addition, we conducted several meta-regression models to assess whether the heterogeneity among controlled studies could be explained by differences in intensity, volume and type of exercise. Similarly, in the analysis that included both controlled and non-controlled studies, we built several linear regression models to assess whether the heterogeneity in the effect measure could be explained by differences in intensity, volume and type of exercise among study arms. These analyses were not included in the original protocol, which was limited to the calculation of correlation coefficients between SMD and the three dimensions of exercise. We later realised that the use of a regression framework instead of correlation, would reveal a clearer picture of the study findings.

A sensitivity analysis was performed by excluding all exercise activities with MET ≤2.5 under the assumption that exercise can only increase BDNF levels through physical conditioning, thus requiring higher intensity (Nofuji et al., 2012; Kelly et al., 2017).

All analyses were done with the statistical software STATA release 18 (StataCorp LP, Texas, USA), while Robvis was used to visualize risk-of-bias assessments (McGuinness and Higgins, 2020).

## Results

### Search results

**FIGURE 1:**
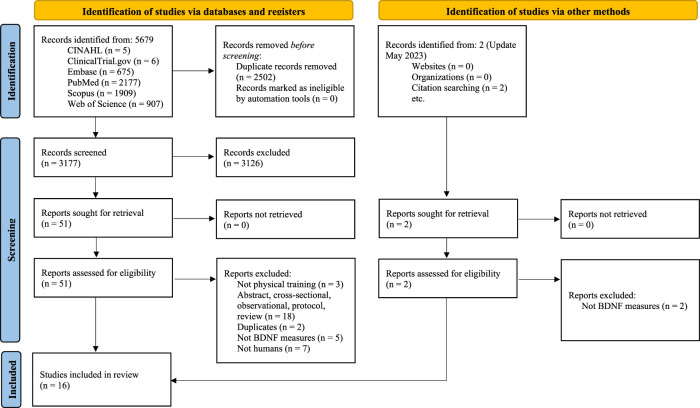
PRISMA 2020 flow diagram for new systematic reviews which included searches of databases, registers and other sources.

A total of 3,179 records were identified after removing duplicates. Fifty-three studies were considered potentially relevant by screening titles and abstracts and 16 studies (8 two-arm trials (Frazzitta et al., 2014; Freidle et al., 2022; Landers et al., 2019; O'Callaghan et al., 2020; Sajatovic et al., 2017; Segura et al., 2020; Soke et al., 2021; Szymura et al., 2020) and 8 single-arm trials (Oliveira et al., 2020; Angelucci et al., 2016; da Silva Germanos et al., 2019; Harro et al., 2022; Ponde et al., 2019; Schaeffer et al., 2022; Stuckenschneider et al., 2021; Zoladz et al., 2014)), which included a total of 370 patients with PD, were finally deemed eligible for the systematic review. The PRISMA flow diagram illustrating the number of studies excluded at each stage of the systematic review is shown in Figure 1. The studies excluded and the reasons for exclusion are reported in Supplementary Material S3.

### Study characteristics

Table 1 contains a summary description of patient characteristics and interventions for each study arm of the selected studies. A large variability emerged in terms of clinical characteristics and exercise interventions. Sample size ranged from 8 to 95 patients and was often smaller than 20 while mean disease duration varied between 8 months and 14 years. About half of the studies did not report data on MDS-UPDRS and only two studies described the amount of daily levodopa (Landers et al., 2019; Freidle et al., 2022). Most of the interventions were aerobic, followed by resistance and balance and then other components.

**TABLE 1:** Patients’ characteristics and description of the exercise intervention by study arm.

Author and year	Age^a^	Hoen and yahr^b^	Years with disease^a^	N. of subjects	Study arm^c^	Description of exercise protocol	Duration (weeks)
Angelucci et al. (2016)	62.8 ± 6.7	2–3	11.8 ± 7.4	9	Multimodal	Relaxation and breathing, flexibility, postural; treadmill with a heart reserve ≤60% and maximum speed of treadmill scrolling of 3.5–4 km/h; stationary bike with maximum speed of 25–30 km/h; Wii Fit Balance Board; mobility (exercises to promote control of strength and movement velocity); coordination (exercises to promote control of strength and movement velocity)	4
da Silva Germanos et al. (2019)	65.1 ± 6.7	1–3	-	9	Multimodal	Aquatic exercises: stretching and mobility; strengthening, gait, balance, proprioception; dual task (exercises in combination with games with playful connotations to stimulate group integration)	4
Oliveira et al. (2020)	65.5 ± 2.2	1–4	-	9	Multimodal	Mobility and coordination (strength, agility, double task and body control); deep water running (endurance) with distance and speed determined in periodization; balance; stretching and relaxation	4
Frazzitta et al. (2014)	67.0 ± 5.0	1–1.5	0.7 ± 0.4	14	Multimodal	Cardiovascular exercises (warm up), stretching, mobility, postural; balance and gait using a stabilometric platform with a visual cue, treadmill training with both a visual and an auditory cue (heart rate reserve ≤60% and a maximum speed of treadmill scrolling of 3.5 km/h); occupational therapy	4
	65 ± 4	1–1.5	0.7 ± 0.2	10	None		
Freidle et al. (2022)	71.0 ± 5.9	2–3	5.5 ± 7	48	Multimodal	HiBalance (sensory integration, motor agility, anticipatory postural adjustments and stability limits); home exercises (functional aerobic, strength exercises)	10
	71.1 ± 6.3	2–4	3 ± 4	47	Speech and communication	HiCommunication (voice sound level, articulatory precision, word retrieval, memory); home exercises (voice, speech function)	10
Harro et al. (2022)	67.2 ± 9.2	1.5–3	4.5 ± 3.2	12	Endurance	Nordic walking: warm up, low intensity aerobic exercise at 4–6/10 RPE with intermittent high intensity short intervals of aerobic exercise at 7–8/10 RPE during training progressions, cool down	6
Landers et al. (2019)	63.5 ± 10.9	1–3	4.9 ± 5.1	13	Multimodal (high intensity)	High-intensity multimodal exercise boot camp (HIBC): moderate-high intensity aerobic exercise at 70%–80% of HRmax (treadmill, overground walking on the indoor track, stair climber, bike, recumbent bike, rowing machine), strengthening the major muscle groups of the trunk and upper/lower extremities at 50–80 1RM, postural, dynamic gait and sensory orientation, stretching; home exercises at 13–20 RPE	8
	64.6 ± 6.0	1–4	4.7 ± 3.9	11	Multimodal (low intensity)	Usual care exercise program (UC): aerobic exercise at 50%–65% of HRmax (treadmill, overground walking on the indoor track, stair climber, bike, recumbent bike, rowing machine), strengthening the major muscle groups of the trunk and upper/lower extremities (no more than 50% of their 1RM), step touch task, stretching; home exercises at 13–20 RPE	8
O'Callaghan et al. (2020)	70.4 ± 7.2	1–3	-	13	Aerobic + Resistance	Moderate-intensity continuous training at 60–80 HRmax: aerobic exercises included warm-up, treadmill walking/running, sit-to-stand, marching/step ups, hand cycling, recumbent cycling and boxing; resistance exercises included upright rowing, lateral arm raises, triceps kick-backs, chest stretch at 90° and bicep curls (circuit with 12 workstations); stretching	12
	64.6 ± 8.6	1–3	-	14	None		
	68.8 ± 7.9	1–3	-	9	High-Intensity Interval Training	High intensity interval training at ≥85% HRmax: warm-up which progressed in intensity and contained whole-body movements (e.g., power clean and press, step and press, squat, pull-down to squat, high pull, bent over row); cool down	12
	69.0 ± 6.6	1–3	-	8	None		
Ponde et al. (2019)	60.6 ± 14.5	2–3	9.4 ± 5.9	8	Aerobic	Aerobic training (treadmill) at 50%–70% HRmax; motor imagery training including concentration with instrumental music, imaginary practice, return of images (video projection) recorded in physical training (real), referring to the running on a treadmill, relaxation	8
Sajatovic et al. (2017)	69.8 ± 2.3	1–3	7.3 ± 3.4	13	Aerobic + Resistance (peer support)	EXCEED (Enhanced ExerCisE thErapy) at 60%–80% HRmax: warm up, fast-paced, low-resistance cycling, progressive sequence of resistance bands, cool down	12
	70.3 ± 6.5	1–3	6.4 ± 6.7	15	Aerobic + Resistance	SGE (Self-guided exercise) at 60%–80% HRmax: warm up, fast-paced, low-resistance cycling, progressive sequence of resistance bands, cool down	12
Schaeffer et al. (2022)	58 ± 10	1–2.5	5.6 ± 5.0	17	Aerobic	Exergame training (“Virtual smash”, “Light race” and “Kardio boxing” from the game pack “Your shape: Fitness evolved”) combined aspects of moderate intensity aerobic fitness training with elements of coordination and speed	6
Segura et al. (2020)	57.8	1–3	2–14	6	Aerobic	Warm up (low resistance pedalling at 30–40 rpm), stationary tandem bicycle at 80% HRmax (while pedalling at 80 rpm or faster), cool down (30–40 rpm), stretching	16
	56	1–3	2–24	7	None		
Soke et al. (2021)	57.1 ± 8.2	1–3	8.1 ± 4.8	15	Aerobic + Resistance	Warm up, treadmill at 60%–80% HRmax, cool down, task-oriented training (TOT): circuit with 11 workstations (12–15 RPE)	8
	58.1 ± 8.9	1–3	7.6 ± 3.9	14	Aerobic	Warm up, treadmill at 60%–80% HRmax, cool down	8
Stuckenschneider et al. (2021)	71.4	1.5–4	-	8	Multimodal	Standing on even or uneven ground with eyes open or closed (balance), sitting on a chair while lifting one’s toes or heels and throwing and catching a ball at the same time (dual task); wall pushups, holding medicine balls with outstretched arms, squats, or crunches (resistance) at least 13 RPE; walking or running exercises (aerobic) at least 13 RPE	8
Szymura et al. (2020)	66.0 ± 2.6	2–3	-	16	Aerobic	Warm up, Wii Fit Balance Board at 60%–70% HRmax, cool down	12
	65.2 ± 7.4	2–3	-	13	None		
Zoladz et al. (2014)	70 ± 3	1–3	8.5 ± 4.5	12	Aerobic	The moderate-intensity interval training session (IT) consisted of warm up, interval exercise including cycling at 80–90 rpm (fast phase of IT) and cycling less than 60 rpm (slow phase of IT) at 60%–75% HRmax, cool down	8

^a^ Mean ± Standard Deviation.

^b^ Range.

^c^ Definition of exercise intervention according to Zhou B et al. in Aging Neurosci 2022 (Zhou et al., 2022).

Supplementary Material S4 details all exercise activities included in each study arm according to type (Aerobic, Resistance, Balance, Others), MET, number of sessions per week and minutes per session. Table 2 shows average intensity, total and weekly volume, and proportion of volume according to exercise type by study arm. Exercise protocols had a median intensity of 3.5 MET (inter-quartile range 3.1–3.9); weekly and total volume were highly variable.

**TABLE 2:** Intensity, weekly and total volume, and proportion of exercise type by study arm.

Author and year	Study arm^a^	Intensity (time-weighted average of MET)	Total volume (MET-hours)	Proportion aerobic	Proportion balance	Proportion resistance	Proportion others
Angelucci et al. (2016)	Multimodal	2.5	123.2	0.32	0.12	0	0.55
Da Silva Germanos et al. (2019)	Multimodal	3.7	24.7	0.32	0	0.54	0.14
Oliveira et al. (2020)	Multimodal	3.9	32.8	0.70	0.16	0	0.14
Frazzitta et al. (2014)	Multimodal	2.4	103.7	0.39	0.12	0	0.49
Freidle et al. (2022)	Multimodal	3.1	73.3	0.16	0.68	0.16	0
	Speech and communication	1.3	28.2	0	0	0	1
Harro et al. (2022)	Endurance	4.2	60.4	1	0	0	0
Landers et al. (2019)	Multimodal (high intensity)	3.8	215.4	0.38	0.13	0.38	0.1
	Multimodal (low intensity)	2.7	90	0.31	0.19	0.33	0.17
O'Callaghan et al. (2020)	Aerobic + Resistance	3.5	123.1	0.72	0	0.23	0.04
	High-Intensity Interval Training	5.3	111	0	0	1	0
Ponde et al. (2019)	Aerobic	2.5	36	0.89	0	0	0.11
Sajatovic et al. (2017)	Aerobic + Resistance (peer support)	3.5	105	0.60	0	0.40	0
	Aerobic + Resistance	3.5	105	0.60	0	0.40	0
Schaeffer et al. (2022)	Aerobic	4	54	1	0	0	0
Segura et al. (2020)	Aerobic	4.3	144.4	0.87	0	0	0.13
Soke et al. (2021)	Aerobic + Resistance	3.9	98.8	0.47	0	0.53	0
	Aerobic	3.8	46	1	0	0	0
Stuckenschneider et al. (2021)	Multimodal	3.6	65.6	0.38	0.23	0.38	0
Szymura et al. (2020)	Aerobic	3.2	114	0.13	0.87	0	0
Zoladz et al. (2014)	Aerobic	3.2	76	1	0	0	0

^a^ Definition of exercise intervention according Zhou B et al. in Aging Neurosci 2022 (Zhou et al., 2022).

### Main analysis

Supplementary Material S5 shows pre-post exercise BDNF levels of experimental and comparison groups with SMD (Hedges’ g) in randomized studies. Only two out of eight controlled studies showed significant differences in BDNF changes over time between experimental and comparison groups.

**FIGURE 2:**
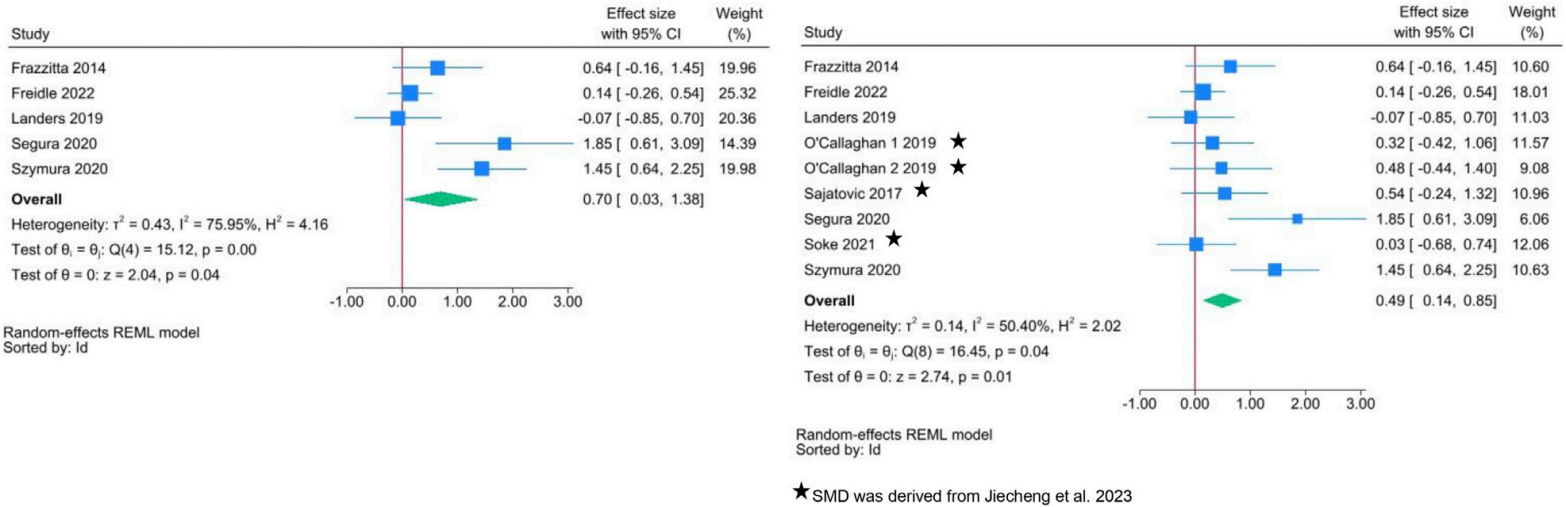
Forest plots of exercise interventions in a subset of 5 controlled studies with available data on BDNF (left) and in the complete set of 9 controlled studies (right).

Two separate meta-analyses were performed (Figure 2): the first one included only those studies (*n* = 5) which reported mean and standard deviation of BDNF, allowing us to directly estimate SMD. The second meta-analysis added data on SMD derived from a previous systematic review (American College of Sports Medicine et al., 2020). According to the first meta-analysis, exercise interventions show a significant improvement in BDNF levels compared to the control group, with SMD = 0.70 (95% CI: 0.03, 1.38). There was substantial heterogeneity between studies (I^2^ = 76.0%). A smaller but more precise estimate of SMD is present in the meta-analysis of all controlled studies.

**FIGURE 3:**
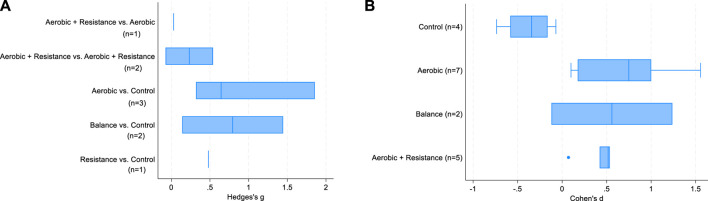
Box-plots of SMD in controlled studies **(A)** and in the analysis by study arms of controlled and non-controlled studies **(B)**.

Table 3 shows the results of the meta-regressions: between-group differences in intensity were positively associated with SMD in the analysis limited to the subset of 5 studies, but not in the complete set of controlled studies. There was no difference according to exercise type. This latter finding is also confirmed in Figure 3A: when the comparison group receives “usual care” (i.e., inactive group), the increase in BDNF change is clear regardless of exercise type. A large variability in SMD is present, especially for aerobic and balance exercise.

**TABLE 3:** Meta-regression of between-group differences in intensity, volume and type of exercise on changes in BDNF levels (controlled studies only).

	5 studies					9 studies		
	Coefficient	(95% CI)		*p*-Value	Coefficient	(95% CI)		*p*-Value
**Δ Time-weighted average of MET^a^**	0.99	(0.10	1.87)	0.028	0.16	(-0.13	0.46)	0.278
**Δ Total MET-hours (x100)^a^**	0.82	(-1.17	2.81)	0.419	0.38	(-0.42	1.18)	0.351
**Type**								
**Identical (Aerobic + Resistance)**	References				References			
**Aerobic**	1.25	(-0.93	3.43)	0.262	0.58	(-0.64	1.79)	0.350
**Balance**	0.81	(-1.29	2.91)	0.449	0.45	(-0.80	1.70)	0.476
**Resistance**					0.00	(-1.31	1.31)	0.999

^a^ Differencebetween experimental and control group.

Supplementary Material S6 shows pre-post exercise BDNF levels with SMD (Cohen’s d) separately by study arm in controlled and non-controlled studies. The SMD was significantly different from zero in 4 study arms. Mean and standard deviation of BDNF were not reported in 4 studies, and the corresponding 8 study arms.

The linear regression models (Table 4) showed that time-weighted average of MET and total volume were positively associated with BDNF change over time. All types of exercise were significantly associated with SMD, with effects of similar magnitude. This finding clearly emerges in Figure 3B as well, which again suggests a substantial effect of exercise on BDNF changes over time regardless of exercise type. A large variability for aerobic and balance exercise is evident.

**TABLE 4:** Linear regression of intensity, volume and type of exercise on change in BDNF (study arms of controlled and non-controlled studies).

	Coefficient	(95% CI)		*p*-Value
**Time-weighted average of MET**	0.38	(0.16	0.59)	0.002
**Total MET-hours (x100)**	0.50	(0.05	0.95)	0.032
**Type**	None as References group			
**Aerobic**	1.10	(0.50	1.70)	0.002
**Balance**	0.93	(0.10	1.77)	0.031
**Aerobic + Resistance**	0.79	(0.14	1.43)	0.020

The results of the sensitivity analysis largely overlapped with those of the primary analysis, with the only relevant difference being the estimate of the effect of exercise intensity (Supplementary Material S7). Time-weighted average of MET was again significant in the meta-regression of 5 controlled studies (Beta coefficient = 0.41, *p* = 0.04) and nearly significant (Beta coefficient = 0.14, *p* = 0.12) in the complete set of controlled studies.

### Risk of bias

The risk of bias assessment for each study is summarized in Supplementary Materials S8, S9. Overall, most controlled studies were at low risk of bias (75%) and the remainder were uncertain. On the other hand, we found a moderate risk of bias for single-arm studies.

## Discussion

This systematic review shows that exercise increases BDNF levels in patients with PD, irrespective of type. Exercises of greater intensity determined the largest improvement. The positive effect of exercise on BDNF levels found in this study confirms the results of previous reviews (Hirsch et al., 2018; Ruiz-Gonzalez et al., 2021; Li et al., 2023; Rotondo et al., 2023). To the best of our knowledge, this is the first systematic review in patients with PD that attempts at evaluating the effect on BDNF levels of different characteristics of exercise, such as intensity, volume and type. A similar analysis was carried out in previous systematic reviews of the literature in youth and athletes (Lesinski et al., 2016; Wu et al., 2021), but not in patients with PD.

As far as intensity of exercise is concerned, our results suggest a positive dose-response association with BDNF levels, resembling the findings of previous reviews in patients with PD (Ellis and Rochester, 2018; Alberts and Rosenfeldt, 2020; Machado et al., 2022), healthy subjects and animal models (Petzinger et al., 2010; Huang et al., 2014). High-intensity exercise may promote the synthesis or availability of BDNF in the brain through different mechanisms, such as increased permeability of the blood brain barrier due to hyperthermia (Walsh and Tschakovsky, 2018), brain hypoxia and muscle damage (Jiménez-Maldonado et al., 2018), and circulating molecules such as lactate (Sobral-Monteiro-Junior et al., 2019).

In the analysis by study arm, total exercise volume was associated with increased BDNF levels over time. A previous study in patients with different neurodegenerative disorders showed that neither weekly volume nor duration of exercise were associated with BDNF (Ruiz-Gonzalez et al., 2021). Since total exercise volume comprehends weekly volume and duration of the intervention, its positive effect on BDNF may be explained by the impact of exercise programs of longer duration. However, total volume was associated with increase in BDNF only in the analysis by study arm, and not in the analysis of controlled studies: it is possible that this positive effect is an artifact due to the high risk of bias of non-controlled studies. De la Rosa et al. reported that trained men have lower circulating levels of BDNF but increased binding sites as a result of an adaptation to regular physical activity (De la Rosa et al., 2019).

The type of exercise that should be recommended to patients with PD is controversial (Li et al., 2023). In this study we found that all types of exercise were useful, but no exercise type was superior to the others, contrary to previous reviews which either favored AT or RT (Dinoff et al., 2016; Zhou et al., 2022). On the other hand, exercises of greater intensity showed the largest benefits in this study. We suggest that patients with mild to moderate disease severity, when appropriately supervised, engage in high-intensity aerobic training (HIIT) or high-intensity resistance training (HIRT) exercises, as recommended by other authors (Zhou et al., 2022; Harpham et al., 2023).

Different mechanisms may explain the effect on BDNF levels of aerobic and resistance exercise, which include increases in Ca^++^ levels and reactive oxygen species in neuronal cells (Radak et al., 2016; Fernandes et al., 2017; Pinho et al., 2019). It was also shown that the release of BDNF from muscle contraction induces the phosphorylation cascades of different signaling pathways, such as cAMP-response element-binding protein (CREB) and mammalian target of rapamycin (mTOR), resulting in additional secretion of BDNF in the brain (Pinho et al., 2019). As far as balance exercise is concerned, the mechanism of action leading to increased BDNF is poorly understood (Kubica et al., 2019).

The present study may be affected by several limitations, such as the scarcity of published studies, most of which had small sample sizes, as well as the large variability in patients’ features and exercise protocols. A recent review on the effect of exercise in PD examined 156 experimental studies (Ernst et al., 2023), but only a minority reported data on BDNF, as shown in this and previous systematic reviews (Hirsch et al., 2018; Li et al., 2023; Rotondo et al., 2023). It is likely that the limited sample size, which was often smaller than 20 patients, combined with between-study patient differences, affected the precision of our estimates. The large heterogeneity in the effect measures, especially for aerobic and balance exercises, may derive from the presence of a few influential studies. Despite the selected studies widely differed in intensity, type and volume, we were able to demonstrate that part of the heterogeneity could be explained by differences in intensity between experimental and control group.

Intensity, volume and typology are not the only dimensions of exercise. Other scholars suggested that “complex” exercise interventions may be particularly effective in patients with PD (Petzinger et al., 2010; Janssen Daalen et al., 2022). These interventions may combine cognitive and motor rehabilitation (Ferrazzoli et al., 2018). Unfortunately, the description of exercise protocols was often poor, so we chose to focus on the dimensions (intensity, volume and type) that were more clearly reported.

Another limitation of this study is the use of 2011 compendium and ACSM tables, which contain reference values of exercise intensity for healthy, and not diseased populations. For instance, it was shown that, for the same relative intensity, the metabolic rate of obese elderly patients with type 2 diabetes was lower than that of healthy subjects (Zanuso et al., 2016). Thus, tabulated values of MET may have not correctly reflected the physiological demands of exercise in patients with PD. In addition, selected studies did not consider those activities which are normally carried out by every patient, including those belonging to “true” control groups: as a result, differences in exercise volume and intensity between the “active” experimental group and the “inactive” control group may have been artificially inflated. Further, most studies did not report the presence of genetic variants, which may either promote BDNF synthesis after AT or interfere with neuroplasticity (Mang et al., 2013; Lemos et al., 2016).

## Conclusion

This systematic review offers consistent evidence that exercise is beneficial for patients with PD, irrespective of exercise typology. The evidence derives from both controlled and non-controlled studies and is especially strong for exercises of greater intensity. This latter finding may be of great value to practitioners for the design and implementation of physical exercise interventions in patients with PD. We suggest that patients with mild to moderate disease severity, when appropriately supervised, engage in high-intensity exercises.

Future studies should improve the description of exercise interventions, carefully detailing their intensity using standardised measures such as MET or % Heart Rate Reserve (American College of Sports Medicine et al., 2020; Rotondo et al., 2023). This will enable researchers to assess more accurately the intensity of the exercise protocol and evaluate its effect on BDNF and other patients’ outcomes.

## Data Availability

The datasets presented in the study can be found in the Supplementary Material S4–S6. The names of the repository and accession number can be found in the Supplementary Material.
