# Supplementary material for: Changes of brain-derived neurotrophic factor (BDNF) levels after different exercise protocols: a systematic review of clinical studies in Parkinson’s disease
Source: Front Physiol. 2024 Feb 20;15:1352305. doi: 10.3389/fphys.2024.1352305 (PMC10912511; doi:10.3389/fphys.2024.1352305)

Changes of Brain-Derived Neurotrophic Factor (BDNF) levels after different exercise protocols: a systematic review of clinical studies in Parkinson's disease

Supplementary File 1. Search strategies

Pubmed

Search: ((Parkinson*[Title/Abstract] OR PD[Title/Abstract]) AND (exercise[Title/Abstract] OR "physical activity"[Title/Abstract] OR training[Title/Abstract] OR sport*[Title/Abstract] OR rehabilit*[Title/Abstract] OR "physical therapy"[Title/Abstract] OR physiotherapy[Title/Abstract]) AND (BDNF[Title/Abstract] OR plastic*[Title/Abstract] OR synap*[Title/Abstract] OR neuro*[Title/Abstract] OR cognit*[Title/Abstract] OR biomarker*[Title/Abstract])) NOT ((rat[Title] OR animal[Title] OR mouse[Title] OR mice[Title]) NOT review[Title]) Filters: from 2003/1/1 - 2022/12/31 Sort by: Most Recent

Scopus

(TITLE ((parkinson* OR pd) AND (exercise OR "physical activity" OR training OR sport* OR rehabilit* OR "physical therapy" OR physiotherapy) AND (bdnf OR plastic* OR synap* OR neuro* OR cognit* OR biomarker*)) OR ABS ((parkinson* OR pd) AND (exercise OR "physical activity" OR training OR sport* OR rehabilit* OR "physical therapy" OR physiotherapy) AND (bdnf OR plastic* OR synap* OR neuro* OR cognit* OR biomarker*)) AND NOT TITLE ((rat OR animal OR mouse OR mice) not AND review)) AND PUBYEAR > 2002 AND PUBYEAR < 2023

Web of Science

(Parkinson* OR PD) AND (exercise OR "physical activity" OR training OR sport* OR rehabilit* OR "physical therapy" OR physiotherapy) AND (BDNF OR plastic* OR synap* OR neuro* OR cognit* OR biomarker*) (Title) or (Parkinson* OR PD) AND (exercise OR "physical activity" OR training OR sport* OR rehabilit* OR "physical therapy" OR physiotherapy) AND (BDNF OR plastic* OR synap* OR neuro* OR cognit* OR biomarker*) (Abstract) not (rat OR animal OR mouse OR mice) NOT review (Title)

ClinicalTrial.gov

(Parkinson* OR PD) AND (exercise OR "physical activity" OR training OR sport* OR rehabilit* OR "physical therapy" OR physiotherapy) AND (BDNF OR plastic* OR synap* OR neuro* OR cognit* OR biomarker*) in Record Title OR (Parkinson* OR PD) AND (exercise OR "physical activity" OR training OR sport* OR rehabilit* OR "physical therapy" OR physiotherapy) AND (BDNF OR plastic* OR synap* OR neuro* OR cognit* OR biomarker*) in Abstract NOT (rat OR animal OR mouse OR mice) NOT review in Record Title - (Word variations have been searched)

Embase

(Parkinson* OR PD) AND (exercise OR "physical activity" OR training OR sport* OR rehabilit* OR "physical therapy" OR physiotherapy) AND (BDNF OR plastic* OR synap* OR neuro* OR cognit* OR biomarker*) in Record Title OR (Parkinson* OR PD) AND (exercise OR "physical activity" OR training OR sport* OR rehabilit* OR "physical therapy" OR physiotherapy) AND (BDNF OR plastic* OR synap* OR neuro* OR cognit* OR biomarker*) in Abstract NOT (rat OR animal OR mouse OR mice) NOT review in Record Title - (Word variations have been searched)

CINHAL

(Parkinson* OR PD) AND (exercise OR "physical activity" OR training OR sport* OR rehabilit* OR "physical therapy" OR physiotherapy) AND (BDNF OR plastic* OR synap* OR neuro* OR cognit* OR biomarker*) in Record Title OR (Parkinson* OR PD) AND (exercise OR "physical activity" OR training OR sport* OR rehabilit* OR "physical therapy" OR physiotherapy) AND (BDNF OR plastic* OR synap* OR neuro* OR cognit* OR biomarker*) in Abstract NOT (rat OR animal OR mouse OR mice) NOT review in Record Title - (Word variations have been searched)

Supplementary File 2. Measurement of BDNF by study

**Author and year**	**Sample**	**Unit**	**Timing of post-exercise BDNF measurement**
Angelucci et al. 2016	Serum	pg/mL	At 8:00-9:00 a.m.
Da Silva Germanos et al. 2019	Plasma	pg/ml	Not specified
De Oliveira et al. 2020	Plasma	ng/mg	At 10-11 a.m.
Frazzitta et al. 2014	Serum	ng/mL	At 7:00 am, away from exercise
Freidle et al. 2022	Serum	pg/mL	Within 2-3 weeks after the end of the intervention[a]
Harro et al. 2022	Serum	ng/mL	On a non-exercise day[a]
Landers et al. 2019	Plasma	pg/mL	Between 48 and 72 hours from active participation in the trial[a]
O'Callaghan et al. 2019	Serum	ng/ml	Not specified
Pondé et al. 2019	Serum	pg/mL	Not specified
Sajatovic et al. 2017	Serum	pg/mL	Not specified
Schaeffer et al. 2022	Serum	ng/mL	Away from the exercise[a]
Segura et al. 2020	Plasma	pg/mL	Not specified
Soke et al. 2021	Serum	pg/ml	At 8-9 am, the day after the end of the intervention
Stuckenschneider et al. 2021	Serum	ng/mL	Not on exercise days[a]
Szymura et al. 2020	Serum	ng/mL	At 8-10 a.m., 2 days after the completion of the programme
Zoladz et al. 2014	Serum	pg/mL	One week after the end of the training[a]

[a]Personal communication from the authors

Supplementary File 3. Studies not included in this review and reason for exclusion

**Authors, year**	**Reason for exclusion**
Angelucci et al., 2015 [1]	Not physical training
Angelucci et al., 2015 [1]	Abstract (duplicate)
Azevedo et al., 2022 [2]	Observational study
Bastioli et al., 2022 [3]	Animals
Bekinschtein et al., 2020 [4]	Review
Biagioni et al., 2018 [5]	Abstract
Campos et al., 2016 [6]	Review
Dias Belchior et al., 2017 [7]	Not BDNF measures
Farshbaf et al., 2016 [8]	Review
Farshbaf et al., 2016 [8]	Review (duplicate)
Ferreira et al., 2018 [9]	Review
Fontanesi et al., 2016 [10]	Not BDNF measures
Franzén et al., 2018 [11]	Protocol
Franzén et al., 2019 [12]	Protocol
Goldberg et al., 2015 [13]	Review
Harper et al., 2019 [14]	Not BDNF measures
Harpham et al., 2023 [15]	Not BDNF measures
Hirsch et al., 2016 [16]	Review
Hooper et al., 2016 [17]	Animals
HU et al., 2005 [18]	Not physical training

Karim et al., 2022 [19]	Measure of BDNF <12h (5-60 min)
Khalil et al., 2017 [20]	Cross-sectional
Marusiak et al., 2015 [21]	Not BDNF measures
Mattson et al., 2004 [22]	Animals
Migdadi et al., 2018 [23]	Abstract
Monteiro-Junior et al., 2015 [24]	Review
Norwitz et al., 2020 [25]	Not BDNF measures
Pagnussat et al., 2018 [26]	Not physical training
Patterson et al., 2022 [27]	Protocol
Radak et al., 2007 [28]	Review
Rothman et al., 2012 [29]	Review
Szymura et al., 2019 [30]	Abstract
Tuon et al., 2014 [31]	Animals
van Wegen et al., 2020 [32]	Protocol
Wang et al., 2022 [33]	Animals
Wu et al., 2011 [34]	Animals
Yang et al., 2014 [35]	Animals

1. Angelucci, F., et al., *A pilot study on the effect of cognitive training on BDNF serum levels in individuals with Parkinson's disease.* Front Hum Neurosci, 2015. **9**: p. 130. https://doi.org/10.3389/fnhum.2015.00130
2. Azevedo, L.V.D., et al., *Acute exercise increases BDNF serum levels in patients with Parkinson's disease regardless of depression or fatigue.* EUROPEAN JOURNAL OF SPORT SCIENCE, 2022. **22**(8): p. 1296-1303. https://doi.org/10.1080/17461391.2021.1922505
3. Bastioli, G., et al., *Voluntary Exercise Boosts Striatal Dopamine Release: Evidence for the Necessary and Sufficient Role of BDNF.* J Neurosci, 2022. **42**(23): p. 4725-4736. https://doi.org/10.1523/JNEUROSCI.2273-21.2022
4. Miranda, M., et al., *Brain-Derived Neurotrophic Factor: A Key Molecule for Memory in the Healthy and the Pathological Brain.* Front Cell Neurosci, 2019. **13**: p. 363. https://doi.org/10.3389/fncel.2019.00363
5. Biagioni, M., et al. *Transcranial Magnetic Stimulation and Aerobic Exercise Increase BDNF-TrkB Signaling in Parkinson's Disease*. in *MOVEMENT DISORDERS*. 2018. WILEY 111 RIVER ST, HOBOKEN 07030-5774, NJ USA.

6. Campos, C., et al., *Exercise-induced neuroprotective effects on neurodegenerative diseases: the key role of trophic factors.* Expert Rev Neurother, 2016. **16**(6): p. 723-34. https://doi.org/10.1080/14737175.2016.1179582
7. Belchior, L.D., et al., *Treadmill in Parkinson's: influence on gait, balance, BDNF and Reduced Glutathione.* Fisioterapia em Movimento, 2017. **30**: p. 93-100. https://doi.org/10.1590/1980-5918.030.S01.AO09
8. Farshbaf, M.J., et al., *Does PGC1 alpha/FNDC5/BDNF Elicit the Beneficial Effects of Exercise on Neurodegenerative Disorders?* NEUROMOLECULAR MEDICINE, 2016. **18**(1): p. 1-15. https://doi.org/10.1007/s12017-015-8370-x
9. Ferreira, R.N., et al., *Neurotrophic Factors in Parkinson's Disease: What Have we Learned from Pre-Clinical and Clinical Studies?* Curr Med Chem, 2018. **25**(31): p. 3682-3702. https://doi.org/10.2174/0929867325666180313101536
10. Fontanesi, C., et al., *Intensive Rehabilitation Enhances Lymphocyte BDNF-TrkB Signaling in Patients With Parkinson's Disease.* Neurorehabil Neural Repair, 2016. **30**(5): p. 411-8. https://doi.org/10.1177/1545968315600272
11. Franzen, E., et al. *Linking neuroplastic effects to behavioral changes after balance training in Parkinson's disease: a study protocol of a randomized controlled trial*. in *MOVEMENT DISORDERS*. 2018. WILEY 111 RIVER ST, HOBOKEN 07030-5774, NJ USA. https://www.mdsabstracts.org/abstract/linking-neuroplastic-effects-to-behavioral-changes-after-balance-training-in-parkinsons-disease-a-study-protocol-of-a-randomized-controlled-trial/
12. Franzén, E., et al., *The EXPANd trial: effects of exercise and exploring neuroplastic changes in people with Parkinson's disease: a study protocol for a double-blinded randomized controlled trial.* BMC Neurol, 2019. **19**(1): p. 280. https://doi.org/10.1186/s12883-019-1520-2
13. Goldberg, A., C.L. Curtis, and J.A. Kleim, *Linking Genes to Neurological Clinical Practice: The Genomic Basis for Neurorehabilitation.* JOURNAL OF NEUROLOGIC PHYSICAL THERAPY, 2015. **39**(1): p. 52-61. https://doi.org/10.1097/npt.0000000000000066
14. Harper, S.A., et al., *Non-Motor Symptoms after One Week of High Cadence Cycling in Parkinson's Disease.* Int J Environ Res Public Health, 2019. **16**(12). https://doi.org/10.3390/ijerph16122104
15. Harpham, C., et al., *Co-Creating a Feasible, Acceptable and Safe Home-Based High-Intensity Interval Training Programme for People with Parkinson's: The HIIT- Home4Parkinson's Study.* Int J Environ Res Public Health, 2023. **20**(9). https://doi.org/10.3390/ijerph20095671
16. Hirsch, M.A., S.S. Iyer, and M. Sanjak, *Exercise-induced neuroplasticity in human Parkinson's disease: What is the evidence telling us?* PARKINSONISM & RELATED DISORDERS, 2016. **22**: p. S78-S81. https://doi.org/10.1016/j.parkreldis.2015.09.030
17. Hooper, P.L., et al., *The central role of heat shock factor 1 in synaptic fidelity and memory consolidation.* Cell Stress Chaperones, 2016. **21**(5): p. 745-53. https://doi.org/10.1007/s12192-016-0709-1
18. Hu, D., et al., *Genetic polymorphisms of brain-derived neurotrophic factor and sporadic Parkinson disease.* Chinese Journal of Clinical Rehabilitation, 2005. **9**(17): p. 196-197. https://pesquisa.bvsalud.org/portal/resource/pt/wpr-409402
19. Karim, A., et al., *Evaluation of Sarcopenia Using Biomarkers of the Neuromuscular Junction in Parkinson's Disease.* J Mol Neurosci, 2022. **72**(4): p. 820-829. https://doi.org/10.1007/s12031-022-01970-7
20. Khalil, H., et al., *The Association Between Physical Activity With Cognitive Function and Brain-Derived Neurotrophic Factor in People With Parkinson's Disease: A Pilot Study.* J Aging Phys Act, 2017. **25**(4): p. 646-652. https://doi.org/10.1123/japa.2016-0121
21. Marusiak, J., et al., *INTERVAL TRAINING-INDUCED ALLEVIATION OF RIGIDITY AND HYPERTONIA IN PATIENTS WITH PARKINSON'S DISEASE IS ACCOMPANIED BY INCREASED BASAL SERUM BRAIN-DERIVED NEUROTROPHIC FACTOR A repeated-measures, case series pilot study.* JOURNAL OF REHABILITATION MEDICINE, 2015. **47**(4): p. 372-375. https://doi.org/10.2340/16501977-1931
22. Mattson, M.P., et al., *Prophylactic Activation of Neuroprotective Stress Response Pathways by Dietary and Behavioral Manipulations.* NeuroRx, 2004. **1**(1): p. 111-116. https://doi.org/10.1602/neurorx.1.1.111
23. Migdadi, H., et al., *Modulating BDNF Activity in Parkinson's Disease: the Impact of Aerobic Exercise and Transcranial Magnetic Stimulation (P5. 076)*. 2018, AAN Enterprises. https://www.aan.com/MSA/Public/events/AbstractDetails/38628
24. Monteiro-Junior, R.S., et al., *We need to move more: Neurobiological hypotheses of physical exercise as a treatment for Parkinson's disease.* Med Hypotheses, 2015. **85**(5): p. 537-41. https://doi.org/10.1016/j.mehy.2015.07.011
25. Norwitz, N.G., et al., *A Ketone Ester Drink Enhances Endurance Exercise Performance in Parkinson's Disease.* Front Neurosci, 2020. **14**: p. 584130. https://doi.org/10.3389/fnins.2020.584130

26. Pagnussat, A.S., et al., *Plantar stimulation in parkinsonians: From biomarkers to mobility - randomized-controlled trial.* Restor Neurol Neurosci, 2018. **36**(2): p. 195-205. https://doi.org/10.3233/rnn-170744
27. Patterson, C.G., et al., *Study in Parkinson's disease of exercise phase 3 (SPARX3): study protocol for a randomized controlled trial.* Trials, 2022. **23**(1): p. 855. https://doi.org/10.1186/s13063-022-06703-0
28. Radak, Z., et al., *Effects of exercise on brain function: role of free radicals.* APPLIED PHYSIOLOGY NUTRITION AND METABOLISM-PHYSIOLOGIE APPLIQUEE NUTRITION ET METABOLISME, 2007. **32**(5): p. 942-947. https://doi.org/10.1139/h07-081
29. Rothman, S.M., et al., *Brain-derived neurotrophic factor as a regulator of systemic and brain energy metabolism and cardiovascular health*, in *BRAIN AND OBESITY*, G. Cizza and K.I. Rother, Editors. 2012. p. 49-63. https://doi.org/10.1111/j.1749-6632.2012.06525.x
30. *Abstract book of the 5th World Parkinson Congress, Kyoto, Japan, June 4–7, 2019.* Journal of Parkinson's Disease, 2019. **9**: p. 1-278. https://content.iospress.com/download/journal-of-parkinsons-disease/jpd199900?id=journal-of-parkinsons-disease%2Fjpd199900
31. Tuon, T., et al., *Physical training prevents depressive symptoms and a decrease in brain-derived neurotrophic factor in Parkinson's disease.* Brain Res Bull, 2014. **108**: p. 106-12. https://doi.org/10.1016/j.brainresbull.2014.09.006
32. van Wegen, E.E.H., et al., *High-Intensity Interval Cycle Ergometer Training in Parkinson's Disease: Protocol for Identifying Individual Response Patterns Using a Single-Subject Research Design.* Front Neurol, 2020. **11**: p. 569880. https://doi.org/10.3389/fneur.2020.569880
33. Wang, T.F., et al., *Inhibition of Nigral Microglial Activation Reduces Age-Related Loss of Dopaminergic Neurons and Motor Deficits.* Cells, 2022. **11**(3). https://doi.org/10.3390/cells11030481
34. Wu, S.Y., et al., *Running exercise protects the substantia nigra dopaminergic neurons against inflammation-induced degeneration via the activation of BDNF signaling pathway.* Brain Behav Immun, 2011. **25**(1): p. 135-46. https://doi.org/10.1016/j.bbi.2010.09.006
35. Yang, J.L., et al., *BDNF and exercise enhance neuronal DNA repair by stimulating CREB-mediated production of apurinic/apyrimidinic endonuclease 1.* Neuromolecular Med, 2014. **16**(1): p. 161-174. https://doi.org/10.1007/s12017-013-8270-x

Supplementary File 4. Type, intensity, frequency and duration of exercise components by study arm

Author and year	Study arm[a]	Activity	Type of activity	MET	Sessions week	Min session
Angelucci et al. 2016	Multimodal	Relaxation, breathing	Others	1.5	5	10
		Flexibility	Others	2.0	5	15
		Mobility	Others	2.3	5	20
		Postural	Others	2.5	5	20
		Treadmill	Aerobic	3.0	5	20
		Stationary bike	Aerobic	3.0	5	20
		Wii Fit Balance Board	Balance	2.3	5	20
		Coordination	Others	2.5	5	25
Da Silva Germanos et al. 2019	Multimodal	Aquatic exercises: stretching, mobility	Others	2.5	2	10
		Aquatic exercises: strengthening, gait, balance, proprioception	Resistance	5.0	2	20
		Aquatic exercises: dual task	Aerobic	3.0	2	20
De Oliveira et al. 2020	Multimodal	Mobility, coordination	Others	2.5	2	8
		Deep water running (endurance)	Aerobic	5.0	2	35
		Coordination	Balance	3.0	2	13
		Stretching, relaxation	Others	2.0	2	8
Frazzitta et al. 2014	Multimodal	Cardiovascular exercises	Aerobic	2.5	5	15
		Stretching, mobility	Others	2.3	5	15
		Postural	Others	2.5	5	15
		Stabilometric platform with a visual cue	Balance	2.5	5	15
		Treadmill training with both a visual and an auditory cue	Aerobic	2.8	5	30
		Occupational therapy	Others	2.0	5	40
Freidle et al. 2022	Multimodal	HiBalance: sensory integration, motor agility, anticipatory postural adjustments and stability limits	Balance	3.0	2	50

		Functional aerobic (home exercises)	Aerobic	3.5	1	20
		Strength exercises (home exercises)	Resistance	3.5	1	20
	Speech and communication	HiCommunication: voice sound level, articulatory precision, word retrieval, memory	Others	1.3	2	50
		Voice, speech function (home exercises)	Others	1.3	1	30
Harro et al. 2022	Endurance	Warm up (nordic walking)	Aerobic	2.5	2	10
		Nordic walking	Aerobic	5.0	2	43
		Cool down (nordic walking)	Aerobic	2.5	2	10
Landers et al. 2019	Multimodal (High intensity)	Treadmill, overground walking on the indoor track, stair climber, bike, recumbent bike, rowing machine	Aerobic	4.5	4	30
		Strengthening the major muscle groups of the trunk and upper/lower extremities	Resistance	4.5	4	30
		Postural, dynamic gait, sensory orientation	Balance	3.0	4	15
		Stretching	Others	2.3	4	15
		Home exercise (same exercises)	Aerobic	4.0	2	18
		Home exercise (same exercises)	Resistance	4.0	2	18
		Home exercise (same exercises)	Balance	3.0	2	9
		Home exercise (same exercises)	Others	2.3	2	9
	Multimodal (Low intensity)	Treadmill, overground walking on the indoor track, stair climber, bike, recumbent bike, rowing machine	Aerobic	2.7	4	15
		Strengthening the major muscle groups of the trunk and upper/lower extremities	Resistance	3.0	4	15
		Step touch task	Balance	2.5	4	10
		Stretching	Others	2.3	4	10
		Home exercise (same exercises)	Aerobic	3.0	2	11
		Home exercise (same exercises)	Resistance	3.0	2	11
		Home exercise (same exercises)	Balance	2.5	2	8
		Home exercise (same exercises)	Others	2.3	2	8
O'Callaghan et al. 2019	Aerobic + Resistance	Warm up	Aerobic	2.5	3	8

		Stretching	Others	2.3	3	2
		12 stations in a circuit	Aerobic	4.0	3	27
		Resistance stations	Resistance	4.0	3	12
		Cool down	Aerobic	2.5	3	8
		Stretching	Others	2.3	3	2
	High-Intensity Interval Training	Warm up	Resistance	3.0	3	10
		Power clean and press, step and press, squat, pull-down to squat, high pull, bent over row	Resistance	7.0	3	20
		Cool down	Resistance	3.0	3	5
Pondé et al. 2019	Aerobic	Treadmill	Aerobic	3.0	2	40
		Motor imagery	Others	1.1	2	14
Sajatovic et al. 2017	Aerobic + Resistance (peer support)	Warm up	Aerobic	2.5	3	5
		EXCEED: fast-paced, low-resistance cycling	Aerobic	4.0	3	20
		EXCEED: progressive sequence of resistance bands	Resistance	3.5	3	20
		Cool down	Aerobic	2.5	3	5
	Aerobic + Resistance	Warm up	Aerobic	2.5	3	5
		SGE: fast-paced, low-resistance cycling	Aerobic	4.0	3	20
		SGE: progressive sequence of resistance bands	Resistance	3.5	3	20
		Cool down	Aerobic	2.5	3	5
Schaeffer et al. 2022	Aerobic	Exergaming	Aerobic	4.0	3	45
Segura et al. 2020	Aerobic	Warm up	Aerobic	2.5	3	10
		Stationary tandem bicycle	Aerobic	6.0	3	20
		Cool down	Aerobic	2.5	3	5
		Stretching	Others	2.3	3	10
Soke et al. 2021	Aerobic + Resistance	Warm up	Aerobic	2.5	3	5

		Treadmill	Aerobic	4.5	3	20
		Cool down	Aerobic	2.5	3	5
		Task-oriented training (TOT): circuit with a series of workstations	Resistance	4.0	3	33
	Aerobic	Warm up	Aerobic	2.5	3	5
		Treadmill	Aerobic	4.5	3	20
		Cool down	Aerobic	2.5	3	5
Stuckenschneider et al. 2021	Multimodal	Coordination	Balance	2.5	2	20
		Wall pushups, holding medicine balls with outstretched arms, squats, or crunches	Resistance	4.1	2	20
		Walking or running exercises	Aerobic	4.1	2	20
Szymura et al. 2020	Aerobic	Warm up	Aerobic	2.5	3	5
		Wii Fit Balance Board	Balance	3.3	3	50
		Cool down	Aerobic	2.5	3	5
Zoladz et al. 2014	Aerobic	Warm up	Aerobic	2.5	3	10
		Interval training	Aerobic	3.5	3	40
		Cool down	Aerobic	2.5	3	10

[a]Definition of exercise intervention according Zhou B et al. in Aging Neurosci 2022
Abbrevations: MET, Metabolic Equivalent of Task.

Supplementary File 5. Pre-post exercise BDNF levels of experimental and comparison group in controlled studies only

Author and year	Study arm[a]	BDNF before the exercise[b]	BDNF after the exercise[b]	SMD[c]	(95% CI)	
Frazzitta et al. 2014	Multimodal	21.6 (3.4)	24.8 (6.4)	0.64	(-0.16	1.45)
	Control	22.9 (4.0)	22.6 (4.3)			
Freidle et al. 2022	Multimodal	38010.8 (7956.7)	37169.4 (5928.3)	0.14	(-0.26	0.54)
	Speech and communication	37805.3 (8044.6)	35945.8 (6208.5)			
Landers et al. 2019	Multimodal (high intensity)	1960.2 (1529.5)	2580.7 (1411.2)	-0.07	(-0.85	0.70)
	Multimodal (low intensity)	960.3 (1482.9)	1697.5 (1368.1)			
O'Callaghan et al. 2019	Aerobic + Resistance	1300.1	1338.0	0.32	(-0.42	1.06)
	Control	1470.3	861.4			
	High-Intensity Interval Training	685.0	783.9	0.48	(-0.44	1.40)
	Control	699.9	627.9			
Sajatovic et al. 2017	Aerobic + Resistance (peer support)			0.54	(-0.24	1.32)
	Aerobic + Resistance					
Segura et al. 2020	Aerobic	20.6 (120.0)	207.1 (120.0)	1.85	(0.61	3.09)
	Control	260.0 (150.0)	149.6 (150.0)			
Soke et al. 2021	Aerobic + Resistance	1397.0	1507.5	0.03	(-0.68	0.74)
	Aerobic	1487.6	1411.2			
Szymura et al. 2020	Aerobic	21.2 (8.4)	30.4 (6.3)	1.45	(0.64	2.25)
	Control	30.1 (8.0)	25.8 (11.7)			

[a]Definition of exercise intervention according Zhou B et al. in Aging Neurosci 2022; [b]Mean (Standard deviation). For O'Callaghan et al. 2019, Soke et al. 2021 the median is reported**;** [c]Standardized Mean Difference (Hedges' g); for the studies by O'Callaghan et al. 2019, Sajatovic et al. 2017, Soke et al. 2021 data was derived from Jiecheng et al. 2023

Supplementary File 6. Pre-post exercise BDNF levels separately by study arm in controlled and non-controlled studies

Author and year	Study arm[a]	BDNF before the exercise[b]	BDNF after the exercise[b]	SMD[c]	(95% CI)	
Angelucci et al. 2016	Multimodal	2188.4 (1718.8)	2356.5 (1666.7)	0.10	(-0.83	1.02)
Da Silva Germanos et al. 2019	Multimodal	493.9	933.3	-		
De Oliveira et al. 2020	Multimodal	0.2 (0.1)	0.4 (0.3)	1.00	(0.02	1.98)
Frazzitta et al. 2014	Multimodal	21.6 (3.4)	24.8 (6.4)	0.61	(-0.15	1.37)
	Control	22.9 (4.0)	22.6 (4.3)	-0.07	(-0.95	0.81)
Freidle et al. 2022	Multimodal	38010.8 (7956.7)	37169.4 (5928.3)	-0.12	(-0.52	0.28)
	Speech and communication	37805.3 (8044.6)	35945.8 (6208.5)	-0.26	(-0.66	0.15)
Harro et al. 2022	Endurance	34.8	32.3	-		
Landers et al. 2019	Multimodal (high intensity)	1960.2 (1529.5)	2580.7 (1411.2)	0.42	(-0.36	1.20)
	Multimodal (low intensity)	960.3 (1482.9)	1697.5 (1368.1)	0.52	(-0.33	1.37)
O'Callaghan et al. 2019	Aerobic + Resistance	1300.1	1338.0	-		
	Control	1470.3	861.4	-		
	High-Intensity Interval Training	685.0	783.9	-		
	Control	699.9	627.9	-		
Pondé et al. 2019	Aerobic	88.8 (111.8)	202.6 (183.4)	0.75	(-0.26	1.76)
Sajatovic et al. 2017	Aerobic + Resistance (peer support)	26.8 (15.6)	90.0 (166.4)	0.53	(-0.25	1.32)
	Aerobic + Resistance	26.8 (15.6)	90.0 (166.4)	0.53	(-0.19	1.26)
Schaeffer et al. 2022	Aerobic	23.9 (7.5)	25.2 (7.3)	0.18	(-0.50	0.85)
Segura et al. 2020	Aerobic	20.6 (120.0)	207.1 (120.0)	1.55	(0.26	2.85)
	Control	260.0 (150.0)	149.6 (150.0)	-0.74	(-1.82	0.35)
Soke et al. 2021	Aerobic + Resistance	1397.0	1507.5	-		
	Aerobic	1487.6	1411.2	-		
Stuckenschneider et al. 2021	Multimodal	33.6 (9.7)	34.3 (11.8)	0.07	(-0.91	1.05)
Szymura et al. 2020	Aerobic	21.2 (8.4)	30.4 (6.3)	1.24	(0.48	1.99)
	Control	30.1 (8.0)	25.8 (11.7)	-0.43	(-1.21	0.35)

Zoladz et al. 2014	Aerobic	10977.0 (2618.9)	14206.0 (4350.9)	0.90	(0.06	1.74)

[a]Definition of exercise intervention according Zhou B et al. in Aging Neurosci 2022; [b]Mean (Standard deviation). For the studies by Da Silva Germanos et al. 2019, Harro et al. 2022, O'Callaghan et al. 2019, Soke et al. 2021 the median is reported. [c]Standardized Mean Difference (Cohen's d)

Supplementary File 7. Sensitivity analysis

Meta-regression of between-group differences in intensity, volume and type of exercise on changes in BDNF levels (controlled studies only)

	5 studies				9 studies			
	**Coefficient**	**(95% CI)**		**P value**	**Coefficient**	**(95% CI)**		**P value**
**Δ Time-weighted average of MET**	0.41	(0.02	0.81)	0.042	0.14	(-0.04	0.32)	0.124
**Δ Total MET-hours (x100)**	-0.26	(-2.23	1.71)	0.797	-0.04	(-0.94	0.87)	0.934
**Type**								
**Identical (Aerobic + Resistance)**	Reference				Reference			
**Aerobic**	1.25	(-0.93	3.43)	0.262	0.58	(-0.64	1.79)	0.350
**Balance**	0.81	(-1.29	2.91)	0.449	0.45	(-0.80	1.70)	0.476
**Resistance**					0.00	(-1.31	1.31)	0.999

Linear regression of intensity, volume and type of exercise on change in BDNF (study arms of controlled and non-controlled studies)

	**Coefficient**	**(95% CI)**		**P value**
**Time-weighted average of MET**	0.26	(0.15	0.38)	0.000
**Total MET-hours (x100)**	0.52	(-0.02	1.06)	0.059
**Type**				
**Aerobic**	1.10	(0.50	1.70)	0.002
**Balance**	0.93	(0.10	1.77)	0.031
**Aerobic + Resistance**	0.79	(0.14	1.43)	0.020

Supplementary File 8. Risk of bias assessment using Cochrane risk of bias tool (RoB)

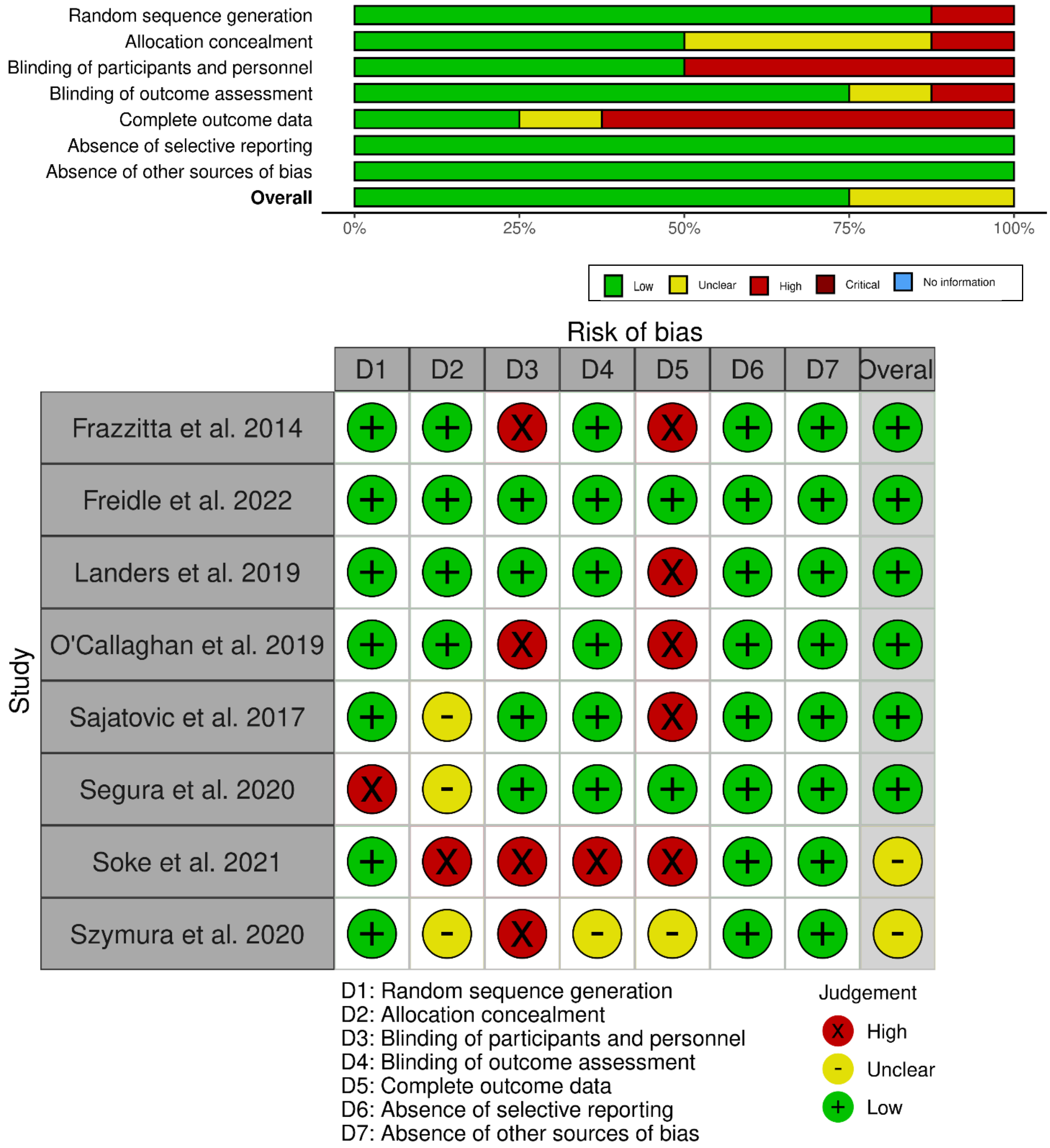

Supplementary File 9. Risk Of Bias In Non-randomized Studies-of Interventions (ROBINS-I)

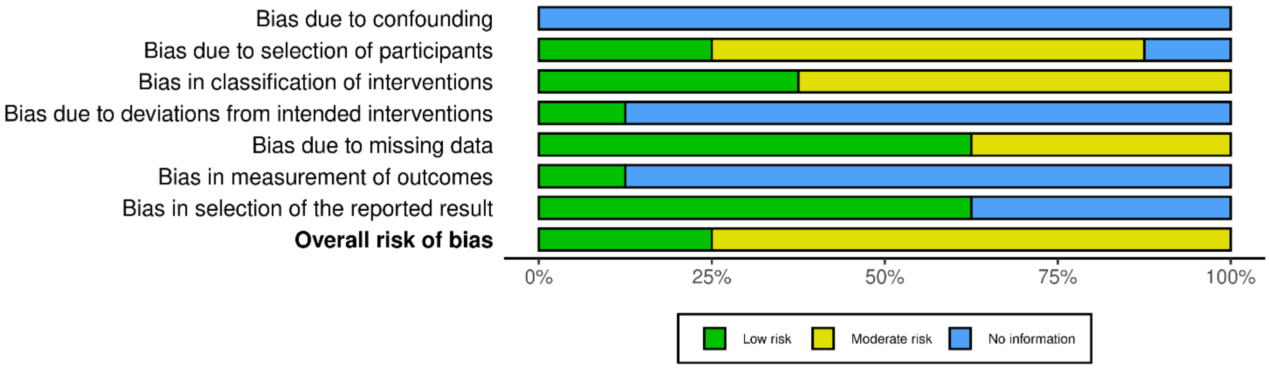

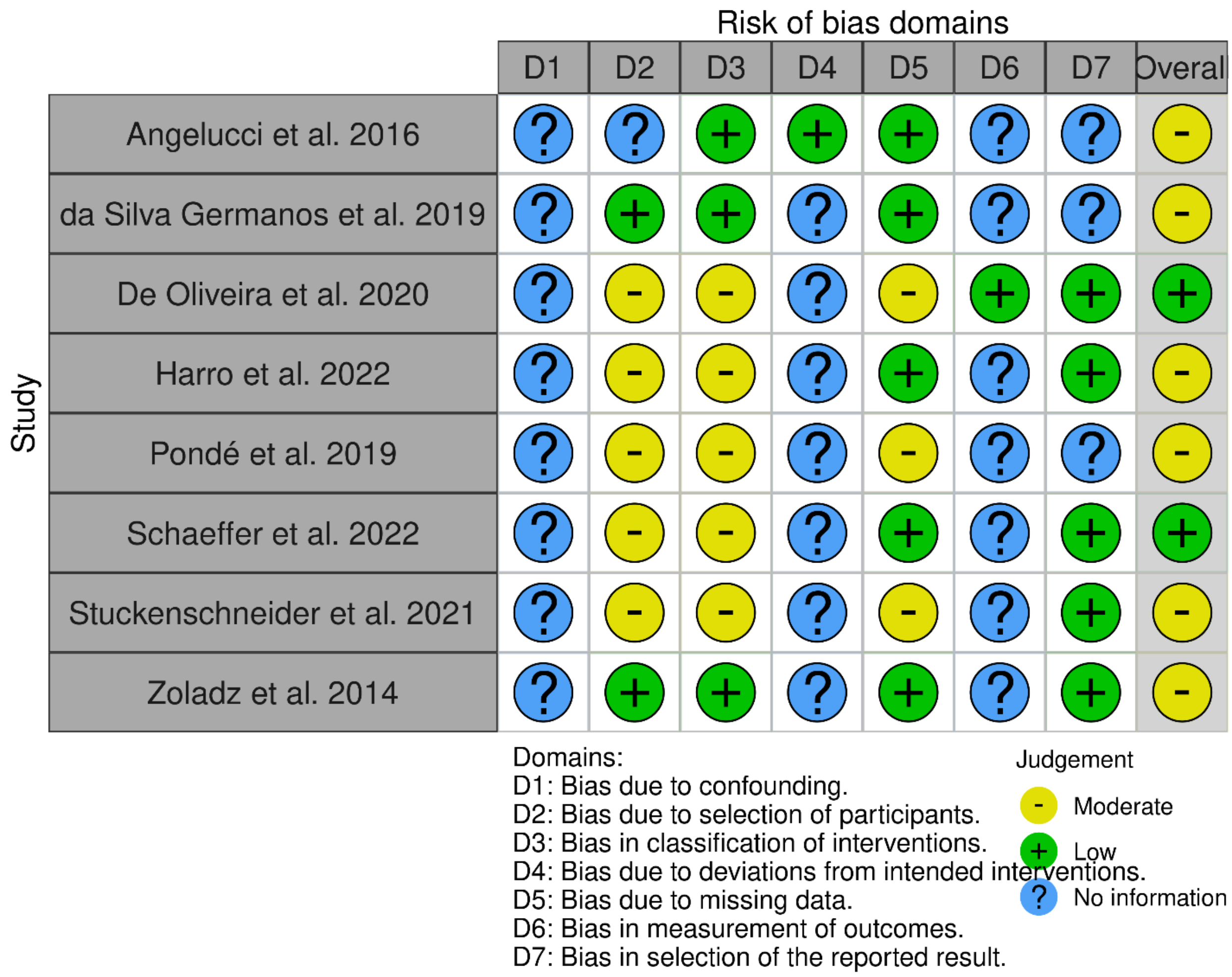

Supplement: Supplementary file 1 [file DataSheet1.pdf]
